# Neural Excitatory/Inhibitory Imbalance in Motor Aging: From Genetic Mechanisms to Therapeutic Challenges

**DOI:** 10.3390/biology14030272

**Published:** 2025-03-07

**Authors:** Xuhui Chen, Ya Wang, Yongning Zhang, Xucheng Li, Le Zhang, Shangbang Gao, Cuntai Zhang

**Affiliations:** 1 Department of Geriatrics, Tongji Hospital, Tongji Medical College, Huazhong University of Science and Technology, Wuhan 430030, China; chenxuhuixy@163.com (X.C.); le_zhang@foxmail.com (L.Z.); 2 Key Laboratory of Vascular Aging, Ministry of Education, Tongji Hospital of Tongji Medical College, Huazhong University of Science and Technology, Wuhan 430030, China; 3 Key Laboratory of Molecular Biophysics of the Ministry of Education, College of Life Science and Technology, Huazhong University of Science and Technology, Wuhan 430030, China; wangya960812@163.com (Y.W.); zhangyongning11@foxmail.com (Y.Z.); tenion2022@163.com (X.L.)

**Keywords:** E/I balance, motor aging, ALS, genetic therapy, excitatory toxicity, inhibitory interneurons

## Abstract

Neural excitatory/inhibitory (E/I) imbalance plays a pivotal role in the aging process. However, despite its significant impact, the role of E/I imbalance in motor dysfunction and neurodegenerative diseases has not received sufficient attention. This review explores the mechanisms underlying motor aging through the lens of E/I balance, emphasizing genetic and molecular factors that contribute to this imbalance (such as *SCN2A*, *CACNA1C*, *GABRB3*, *GRIN2A*, *SYT*, *BDNF*…). Key regulatory genes, including *REST*, *vps-34*, and *STXBP1*, are examined for their roles in modulating synaptic activity and neuronal function during aging. With insights drawn from ALS, we discuss how disruptions in E/I balance contribute to the pathophysiology of age-related motor dysfunction. The genes discussed above exhibit a certain association with age-related motor neuron diseases (like ALS), a relationship that had not been previously recognized. Innovative genetic therapies, such as gene editing technology and optogenetic manipulation, are emerging as promising tools for restoring E/I balance, offering hope for ameliorating motor deficits in aging. This review explores the potential of these technologies to intervene in aging-related motor diseases, despite challenges in their direct application to human conditions.

## Simple Summary

The motivation behind this review stems from the growing understanding that excitatory/inhibitory (E/I) imbalance is a key mechanism in both motor aging and neurodegenerative diseases, such as amyotrophic lateral sclerosis (ALS). By identifying the genetic mutations that contribute to this imbalance, this research aims to offer new insights into how motor function declines with age and disease. Given the difficulty of addressing motor aging at its root, we attempt to propose new approaches, such as gene interventions, to tackle these challenges. This work is timely, as it provides a deeper understanding of the molecular basis of motor aging and suggests therapeutic avenues that could potentially lead to breakthroughs in treatment.

## 1. Introduction

Motor aging, characterized by a decline in movement precision, coordination, and strength, has been linked to alterations in the neural circuits that govern motor function [1]. A crucial aspect of motor system function is the excitatory/inhibitory (E/I) balance, which ensures synaptic integration, neural plasticity, and proper motor control [2]. The E/I balance reflects the interplay between excitatory inputs, predominantly mediated by glutamatergic signaling, and inhibitory inputs, primarily facilitated by GABAergic neurotransmission in the mammalian brain [3]. This balance is affected by the organism’s lifespan as well as by various genetic factors. A significant amount of research has focused on adaptation to reduced neural activity in the context of in vivo plasticity regulation [4]. With aging, a general trend has gradually been established, which is that the percentage of overactive cell components in the body gradually increases, while the percentage of inhibitory cells decreases [5,6]. This trend suggests that inhibitory signaling in the brain cortex may serve as a protective mechanism against neuronal overexcitation [7,8]. However, the protective mechanism may become progressively impaired during the aging process [4,5]. These changes in the nervous system consequently disrupt the fine balance between excitation and inhibition [4], leading to the control failure of motor balance and an increased risk for various motor-related disorders. It has been shown that these changes are conserved in human, mice [4], *C. elegans* [9], and even other organisms [7].

In aged animals, the gradual weakening of inhibitory signaling leads to a decline in cognitive and motor function, further highlighting the critical role of E/I balance in the damage to inhibitory neurons during aging [10,11]. Emerging studies have uncovered age-associated alterations in specific genes (such as *REST* [12], *vps-34* [13], *STXBP1* [14]) involved in synaptic transmission, structural maintenance, and cellular homeostasis. These genes encode diverse proteins, whose mutations disrupt E/I balance at multiple levels, leading to motor dysfunction and an increased risk of neurodegenerative diseases.

Traditionally, amyotrophic lateral sclerosis (ALS) was considered a motor dysfunction disorder caused by a range of genetic mutations, with no clear correlation to age and an early onset in patients [15,16]. However, more recent findings have dramatically shifted this perspective [17]. Studies now show that ALS-related mutations, such as SOD1, impair mitochondria-derived vesicle formation by inducing oxidative stress, a process that accelerates cellular aging [18]. This crucial discovery links ALS to the broader concept of aging-related motor dysfunction and opens new avenues for potential gene therapies targeting aging-associated processes [19]. These insights lay a promising foundation for developing targeted treatments to address both ALS and motor aging, an area that has been largely overlooked in the past.

The innovations of this paper are highlighted in the following aspects: (1) The relationship between neurodegenerative diseases and motor aging is explored from the perspective of E/I imbalance, suggesting that this imbalance may be the key link between the two. (2) Genetic mutations have a profound impact on motor aging, with the paper providing an in-depth analysis of how specific genetic variations accelerate the decline in motor functions, offering a new perspective for future studies on the mechanisms of motor aging. (3) ALS, as a disease closely related to motor aging, is gradually gaining attention. The paper examines the connection between ALS and motor aging in detail, emphasizing the promising research prospects in this field. (4) This review also proposes novel therapeutic approaches, including gene editing and optogenetics, to intervene in motor aging-related diseases, although their direct application to human conditions remains a significant challenge.

## 2. The Definition of Neural E/I Balance

**Figure 1:**
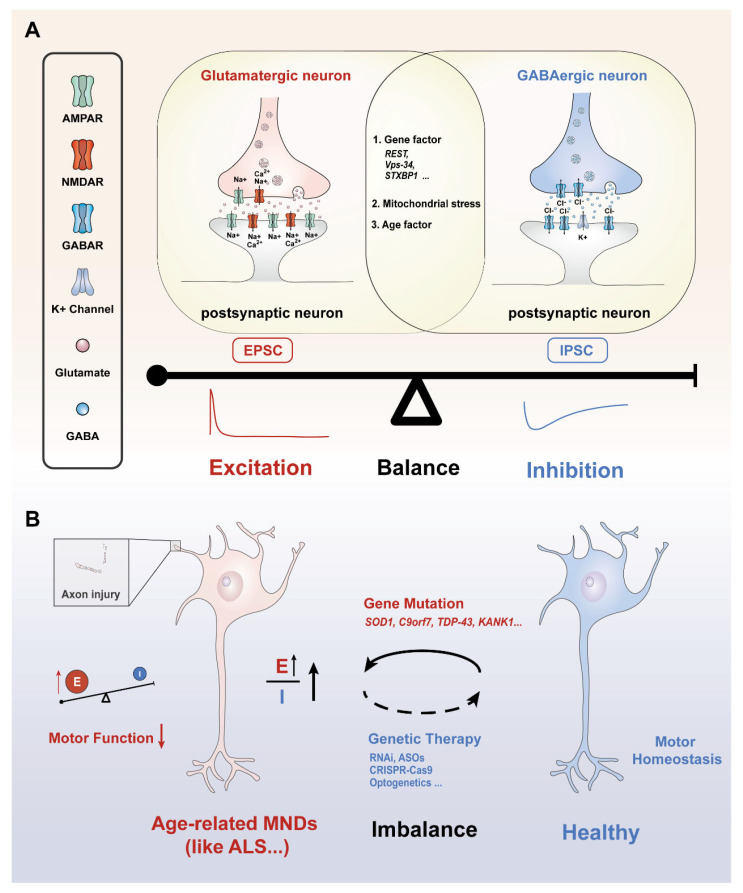
Various factors contribute to excitation/inhibition balance or imbalance. (**A**) Molecular mechanisms and influencing factors underlying the E/I balance: presynaptic glutamatergic and GABAergic neurons release glutamate and GABA into the synaptic cleft, respectively. This process causes postsynaptic neuron excitation or inhibition activity through the flow of ions inside and outside the cell, influenced by genetic factors, mitochondrial stress, and age. (**B**) Age-related MNDs are characterized by an abnormal increase in neuronal excitability, leading to an imbalance between excitatory and inhibitory neurotransmission. This disruption is closely linked to gene mutations, often marked by an increase in excitatory signaling, further resulting in a pathological elevation of the E/I ratio. E/I imbalance could damage axons, impair cellular communication, and disrupt motor function over time. Current gene editing technology or optogenetics may be a promising way to restore motor homeostasis and make motor neurons healthier. Upward Arrow: indicates increase/higher. Downward Arrow: indicates decrease/lower. Red font: excitability-related factors and results. Blue font: inhibition-related influencing factors and results. E: excitability; I: Inhibition.

The balance of neural excitation and inhibition, also referred to as the E/I balance, is a critical aspect of neuronal circuit function, influencing information processing, network stability, and overall brain health [20]. This balance is maintained by fine-tuned excitatory and inhibitory synaptic inputs (Figure 1A) [21]. These inputs are commonly measured by excitatory postsynaptic currents (EPSCs) and inhibitory postsynaptic currents (IPSCs), respectively. EPSCs are the currents that occur when excitatory neurotransmitters (such as glutamate, acetylcholine) bind to postsynaptic receptors (like AMPA and NMDA receptors or AChRs), which essentially release positively charged ions (e.g., Na^+^ and Ca^2+^) into the postsynaptic cells (neurons, muscles and glands) [22]. In neurons, this process causes depolarization of the cellular membrane, making it more likely for the neuron to reach the threshold for generating action potentials. IPSCs are inhibiting currents that result when inhibitory neurotransmitters (such as GABA) bind to postsynaptic receptors (such as GABA_A_ receptors) [23], which are essentially negatively charged ions (e.g., Cl^−^) or the efflux of positively charged ions (e.g., K^+^) (Figure 1A). The excitatory/inhibitory (E/I) balance represents a dynamic equilibrium that ensures optimal neuronal function through the precise regulation of excitatory and inhibitory inputs [24]. Maintaining proper E/I balance is fundamental to normal brain function, while disruptions in this balance are implicated in a wide range of neurological and psychiatric disorders [20,24]. For example, neuronal hyperexcitability, reflected by an increased EPSC-to-IPSC ratio, has been associated with conditions such as epilepsy [25], anxiety disorders [26], and autism spectrum disorders [3]. Conversely, excessive inhibition, characterized by a predominance of IPSCs over EPSCs, results in neuronal hypoactivity. Chronic hypoactivity adversely affects brain functions and has been linked to aging, cognitive impairments [27], and motor neuron diseases [28].

Therefore, the expression of various receptors, neurotrophic factors and vesicle-release-related genes determine various roles for the genesis of network oscillations and the fine-tuning of neuronal circuits (Table 1). It reveals the major facet of homeostatic synaptic plasticity and contributes to the E/I balance under physiological and pathological challenges [29,30,31]. The E/I balance is also altered by genetic background, environment, and age.

## 3. Genetic and Molecular Mechanisms of Motor Aging Underlying the E/I Balance

Aging and many diseases are mutually causal. Compared to younger organisms, the nervous system in aging individuals undergoes a series of changes, including aberrant presynaptic development and dysregulated expression of postsynaptic receptors [4]. For instance, motor neurons in aged systems show synaptic vesicle fusion defects at the neuromuscular junction, followed by abnormalities in quantal size and deficiencies in vesicle docking and priming at a later age [46]. All of these can cause E/I imbalance. Liu et al. were the first to demonstrate the relationship between the decline in motor function and aging in *C. elegans*. Specifically, the deterioration of motor neuron function precedes the disruption of muscle function [46]. In the early stages of aging, the number of receptors compensatorily increases in the muscles in order to offset the reduced input from motor neuron signals [46]. However, this compensation is temporary. As aging advances, the ability of the muscle to maintain this compensation becomes increasingly insufficient [47]. These structural and functional alterations disrupt neurotransmitter release and receptor binding, ultimately impairing synaptic transmission. From an electrophysiological perspective, aging is associated with a decline in excitatory postsynaptic potentials (EPSPs) and an even more pronounced reduction in inhibitory postsynaptic potentials (IPSPs), leading to an elevated E/I ratio [13]. One potential mechanism underlying this phenomenon is the decreased proportion of GABAergic input received by downstream neurons [6,7,9]. Despite GABAergic inhibitory neurons comprising only 10–25% of cortical and hippocampal neuronal populations, their functional decline during aging has an outsized impact on synaptic plasticity, limbic system activity, and neural synchrony [48]. This imbalance disrupts the capacity of downstream neurons to efficiently integrate and process signals from upstream neurons, impairing the precision of neuronal communication [46]. Consequently, these deficits contribute to compromised motor control and coordination, further emphasizing the critical role of E/I balance in maintaining motor function and neural network stability in aging [46].

Moreover, aging influences the neural E/I balance through a wide array of mechanisms, encompassing changes in neuronal population, neural network architecture, neurotransmitter ratios, receptor expression profiles, and receptor levels. These multifaceted alterations collectively modulate synaptic and network-level dynamics, often exacerbating E/I imbalance. Taking into account the latest research advances and our focus, this article highlights several important molecular mechanisms that have been recently discovered (Table 2).

### 3.1. REST

Repressor element-1-silencing transcription factor (REST) is a critical negative regulator of protein transcription with significant roles in aging and neuronal protection [49,50]. Notably, REST levels in the nuclei of prefrontal cortex neurons in centenarians are nearly twice as high as those in individuals aged 70–80, which may contribute to their extended longevity [12,50]. The activation of REST is partly driven by non-autonomous Wnt signaling in cells during aging [51,52]. Conditional knockout of *REST* in the mouse brain leads to increased synaptic dysfunction, neural excitability, and hyperactivity, therefore disrupting the balance of E/I. Interestingly, *REST*-KO mice display age-related neurodegeneration, highlighting the neuroprotective role of *REST* via maintaining E/I balance [52].

REST contributes to E/I balance through its epigenetic regulation of excitatory genes, recruiting histone deacetylases (HDACs) and methylation enzymes to promote long-term silencing [53]. These chromatin modifications provide a robust mechanism for maintaining neural stability across the lifespan. In addition, REST downregulates key proteins essential for presynaptic vesicular release, such as synaptosome-associated protein-25, synaptotagmin-2, and synapsin-1 (Table 1), which helps prevent overactivation of synaptic transmission and excessive neuronal excitation [54,55]. This downregulation further contributes to the protective effects of REST on neural function during aging.

The role of REST in neural protection is closely linked to its function in responding to oxidative stress during aging. The deterioration of oxidative stress homeostasis with age induces an adaptive response involving sustained REST expression, along with increased Akt and decreased mTOR expression [56]. This regulatory shift supports stress resistance mechanisms aimed at preserving neuronal survival and function. REST activation may help mitigate the damaging effects of overexcitation by reducing excitatory activity and increasing levels of key antioxidants, such as superoxide dismutase (SOD), which play a critical role in neutralizing oxygen species (ROS) and maintaining neuronal homeostasis [52]. Overexpression of REST has been shown to upregulate SOD levels, which helps protect against oxidative stress induced by excessive excitatory transmission, particularly in regions prone to neurodegenerative damage, such as the hippocampus [52]. In Alzheimer’s disease (AD), REST expression is diminished in regions of extensive oxidative damage, suggesting a protective role of REST in mitigating the harmful effects of excitatory transmission during aging. Notably, treatment with NMDAR and AMPA receptor antagonists significantly increases global FOXO1 levels in mouse cortical cultures, highlighting a potential link between REST and longevity-associated transcription factors that modulate aging and oxidative stress [12,57,58].

Recent studies have also shown that chronic hyperactivity in primary neuronal cultures induces a globally reduced excitable state, with REST playing a crucial role in mediating this change. About a 75% reduction in Nav1.2 channel density was observed in response to REST activation, as Nav1.2 channels are major targets of REST and are key determinants of membrane excitability [59]. These reductions were accompanied by a decrease in action potential (AP) firing rate, amplitude, and calcium transient spike amplitude, all of which were restored when REST expression was inhibited using shRNA [59]. These findings suggest that REST primarily acts as a compensatory mechanism to counteract E/I imbalance and prevent the harmful effects of chronic hyperexcitation. Supporting this, Pecoraro et al. observed that REST mediates the synaptic homeostatic control of vesicular glutamate transporter-1 density in response to induced hyperexcitability, highlighting its role in stabilizing synaptic function under stress conditions [55].

The neuroprotective function of REST is further supported by studies in animal models. For instance, Dallagnol et al. demonstrated that REST levels are upregulated in the hippocampus of physically active mice compared to their sedentary counterparts, suggesting that physical activity may enhance REST expression and contribute to improved neuronal function with age [60]. In *C. elegans* models, *REST* orthologues, *spr-3* and *spr-4*, regulate E/I balance through the insulin/IGF-1 signaling pathway, with DAF-16 serving as a downstream target [12,58,61,62]. Overexpression of SPR-4 has been linked to reduced neural excitability in the sensory neuron ASH, a phenomenon that contributes to the stability of neural circuits and extended lifespan [12]. Reduced excitation and upregulated REST expression may activate longevity-associated transcription factors like FOXO1 in mammals and DAF-16 in *C. elegans*, thereby mitigating excitotoxicity and delaying aging [12,58].

Therefore, increasing REST levels and reducing excitatory neuronal activity may represent a potential strategy for slowing human motor aging [63]. Through its regulatory effects on excitatory activity, oxidative stress homeostasis, and synaptic plasticity, REST provides a robust neuroprotective mechanism that mitigates the detrimental effects of aging and supports neural function across the lifespan (Table 2).

### 3.2. VPS-34

*Vps-34*, orthologous to human *PIK3C3*, encodes type III phosphatidylinositol 3-kinase (PI3K) kinase, mainly expressed in the cytoplasm and nucleus [64]. VPS-34 is abundantly expressed in the human brain [65] and neuromuscular junctions (NMJs) of *C. elegans* [66]. Research has shown that VPS-34 phosphorylates phosphatidylinositol (PI) phosphate to phosphatidylinositol 3-phosphate (PI(3)P), a key lipid in membrane dynamics [66]. VPS-34 is a central component in endosomes that regulates endocytosis (transport of vesicles and membranes) and autophagy [66,67,68]. These processes are essential for cellular maintenance and synaptic function [68]. With aging, the efficiency of VPS34-mediated autophagic degradation declines, which is linked to neurodegenerative diseases [69]. Autophagy is also involved in synaptic signaling, as it influences the trafficking and endocytosis of synaptic vesicles and proteins [68].

Studies have demonstrated the upregulation of GABAergic signaling at postsynaptic sites in the hippocampal and neocortical neurons of Beclin1-deficient mice [65], closely resembling the effects of SAR405, a selective autophagy inhibitor, on inhibitory transmission in the amygdala [70]. It is suggested that autophagy mainly regulates inhibitory synaptic transmission, particularly by modulating GABA_A_ receptor trafficking through the Beclin1-VPS34-GABARAP cascade [65,71]. This pathway is closely associated with impaired autophagic effects during aging, leading to the downregulation of age-related inhibitory synaptic transmission [65]. In motor neurons, VPS-34 influences synaptic vesicle exocytosis. VPS-34 deficiency can promote exocytosis by promoting PI(3)P-PI-PI(4)P conversion to increase the release of synaptic vesicles in aged worms and mice [13,72,73]. These vesicles store a large number of neurotransmitters and enable communication between neurons and muscles. More interestingly, *vps-34* mutants significantly mitigate the decline in MNs’ electrical activity during aging, and SAR405 has demonstrated potential in reversing these changes [13] (Table 2).

Despite these promising findings, the precise mechanisms by which excitation and inhibition signals are separately affected remain unclear. The role of VPS-34 in motor control within the mammalian brain is still under investigation. Its expression in motor neurons suggests a potential involvement in central motor regulation, and the precise mechanisms remain to be fully elucidated. However, it does not affect *vps-34* as a viable target for treating age-related conditions, with a focus on managing synaptic vesicle exocytosis and maintaining muscular and neuronal function [74]. Exploring its potential impact and E/I homeostasis could yield new insights into managing neural and muscular aging.

### 3.3. STXBP1

STXBP1 (Syntaxin-Binding Protein 1) plays a pivotal role in synaptic vesicle fusion and neurotransmitter release by binding to syntaxin-1, a key synaptic fusion protein, to regulate synaptic secretion [75]. This process is further facilitated by Munc13-1, which helps position synaptic vesicles closer to the active zone of the presynaptic membrane [76]. Munc13-1 enhances the accessibility of syntaxin-1 within the SNARE network motif, thereby optimizing the interaction between STXBP1 and syntaxin-1 for efficient vesicle docking and fusion [76,77].

*STXBP1* haploinsufficiency disrupts cortical inhibitory neurotransmission through two distinct mechanisms involving major GABAergic interneurons: it reduces the synaptic strength of parvalbumin-expressing (PV) interneurons and the connectivity of somatostatin-expressing (SST) interneurons [14]. These disruptions likely contribute to cortical hyperexcitability and the associated neurobehavioral phenotypes observed in STXBP1-related disorders [78]. The differential impact on PV- and SST-mediated inhibition underscores the synapse-specific roles of STXBP1, emphasizing the importance of studying synaptic diversity and specificity in diseased neural circuits [14]. These studies also established STXBP1 mouse models to study *STXBP1* encephalopathy (Table 2).

*STXBP1* haploinsufficiency could cause cortical hyperexcitability and motor and cognitive dysfunction in the elderly [14,79,80]. Motor impairments observed in these disorders may be linked to disruptions in synaptic plasticity, particularly in circuits that control motor coordination [79]. BDNF could upregulate the expression of STXBP1 in neurons to enhance synaptic plasticity and promote neurogenesis [81,82]. Therefore, STXBP1-related modulation of the E/I balance may improve vesicle dynamics and restore synaptic homeostasis, potentially countering the oxidative stress and mitochondrial dysfunction associated with aging. This connection offers a promising strategy for addressing age-related neural impairments while maintaining E/I homeostasis.

## 4. E/I Imbalance in Aging and Motor Dysfunction: Insights from ALS

ALS is a neurodegenerative disorder marked by the degeneration of upper and lower motor neurons, resulting in progressive muscle weakness, atrophy, and paralysis [83,84,85]. ALS was traditionally viewed as an early-onset motor disorder. However, emerging evidence underscores its strong association with aging-related motor dysfunction [18,86]. Epidemiologically, ALS incidence peaks between ages 50 and 70, with cases being rare before age 40 [87]. This suggests that aging significantly enhances susceptibility to ALS. Intriguingly, unlike Alzheimer’s or Parkinson’s disease, ALS incidence declines after the eighth decade of life, likely due to the death of genetically or environmentally susceptible individuals rather than a reduced risk with aging [87,88,89]. This age-dependent pattern reinforces the notion that aging plays a critical role in modifying ALS pathogenesis.

A major contributor to ALS progression is excitatory toxicity, driven by an overactive glutamatergic system, which exacerbates neuronal stress and increases the susceptibility of motor circuits to neuron degeneration [90] (Figure 1B). Notably, aging amplifies glutamate receptor sensitivity and impairs astrocytic glutamate clearance, creating a “storm” for excitotoxic damage in vulnerable motor neurons. This imbalance in excitatory and inhibitory signaling is further exacerbated by glial dysfunction in ALS [91]. Specifically, astrocytic degeneration reduces the expression of the glutamate transporter EAAT2/GLT-1, disrupting synaptic glutamate clearance and prolonging EPSCs [92]. Activated microglia in ALS release inflammatory cytokines such as TNF-α and IL-1β, which increase the surface expression of Ca^2+^-permeable AMPA receptors, amplifying excitatory currents [93,94]. These interconnected glial dysfunctions ultimately intensify excitatory toxicity and motor neuron vulnerability, accelerating the progressive motor dysfunction seen in ALS, particularly in aging individuals.

The introduction of a machine learning model called RefMap has revolutionized genetic research in ALS. RefMap integrates GWAS data with transcriptomic and epigenetic profiling of induced pluripotent stem cell-derived motor neurons. RefMap has identified 690 ALS-associated genes, representing a five-fold increase in heritability compared to traditional methods [95]. Among the genes identified, *KANK1* emerged as a novel and significant risk factor for ALS. *KANK1* is functionally related to other known ALS genes critical for cytoskeletal function, such as *PFN1*, *KIF5A*, and *TUBA4A*. Similar to *KANK1*, *PFN1* plays a role in actin polymerization, a process vital for maintaining synaptic organization and proper nucleocytoplasmic transport [96]. Actin polymerization might closely link to synaptic structure and function, including the formation and maintenance of dendritic spines, which are primary sites of excitatory synaptic input. Disruption of this process may impair NMJs and synaptic stability, leading to an imbalance in E/I inputs, thereby contributing to motor neuron hyperexcitability often observed in ALS [97]. Mutations in *KANK1* were shown to disrupt axonal function and cause TDP-43 mislocalization, a hallmark of ALS pathology [95]. These findings link *KANK1* mutations with earlier disease onset and reproducing key ALS phenotypes in neuronal models.

TDP-43, a hallmark protein of ALS, exacerbates aging-related neuronal degeneration by triggering mitochondrial DNA (mtDNA) release through the mitochondrial permeability transition pore (mPTP) [98]. This process activates the cGAS/STING inflammatory pathway, leading to neuroinflammatory responses characterized by elevated nuclear factor kB (NF-kB) and type I interferon signaling [99]. Elevated cGAMP levels, a marker of cGAS/STING activation, were detected in ALS patient samples, indicating the pathway’s involvement in disease progression [99]. Age-related declines in mitochondrial repair exacerbate these effects, as aging neurons are less equipped to mitigate mtDNA stress and inflammation. Mitochondrial dysfunction in ALS may also directly exacerbate neuronal hyperexcitability through multiple mechanisms [100]. For instance, impaired mitochondrial ATP production compromises ion pump activity (e.g., Na+/K+-ATPase), disrupting resting membrane potential regulation. Concurrently, defective mitochondrial calcium buffering elevates cytosolic calcium levels, which could amplify excitotoxic cascades—a hallmark of ALS pathology [101,102]. Furthermore, aging amplifies the impact of TDP-43 mitochondrial localization, leading to increased production of reactive oxygen species (ROS) and reduced mitochondrial membrane potential [98,103]. These factors heighten the vulnerability of aging neurons, making them more susceptible to TDP-43-induced damage.

Beyond its mitochondrial effects, TDP-43 also disrupts neuronal excitability. Recent studies have shown that TDP-43’s effects on the axon initial segment (AIS), the site where action potentials are generated, significantly contribute to this dysregulation [104,105]. In early ALS stages, such as in human iPSC-derived motor neurons from patients with *TDP-43* and *C9orf72* mutations, there is an increase in AIS length, as well as impaired activity-dependent AIS plasticity [105,106]. This disruption is linked to abnormal homeostatic regulation of neuronal activity and intrinsic hyperexcitability, where neurons exhibit increased spontaneous firing [105]. These hyperactive neurons, in turn, contribute to the pathological symptoms of ALS, such as increased spontaneous myofiber contractions in vitro. Interestingly, this hyperexcitability in early ALS stages contrasts with later-stage hiPSC motor neurons and postmortem ALS neurons, which show AIS shortening and progression to hypoexcitability [105]. At the molecular level, these dynamic changes in AIS structure and function correlate with altered expression of critical AIS scaffolding proteins like ankyrin-G, as well as voltage-gated sodium channels specific to the AIS [105]. These molecular disruptions amplify TDP-43’s impact on the E/I balance, making neurons more prone to dysfunction.

Growing evidence suggests that key genes implicated in the regulation of age-related ALS surprisingly exhibit significant disruption of the E/I balance at both the synaptic and circuit levels. These striking findings prompt a critical question: can E/I balance serve as a benchmark for identifying innovative interventions or even therapeutic strategies for aging-related motor diseases?

## 5. Emerging Genetic Therapies for Restoring E/I Balance

### 5.1. Gene Editing Technology

Gene editing technology, such as RNA interference (RNAi), antisense oligonucleotides (ASOs) [107,108], and the CRISPR-Cas9 system [109,110,111], could target and suppress the expression of mutant genes contributing to neurodegeneration. These technologies highlight the potential for treating E/I imbalance in motor aging from a genetic perspective. For example, in ALS caused by *SOD1* mutations, RNAi selectively silences the mutant allele, reducing excitotoxic protein aggregates and improving motor neuron survival [112]. Building on this approach, the FDA granted accelerated approval in April 2023 for Qalsody (tofersen), an ASO therapy designed to target *SOD1* mRNA and reduce toxic protein synthesis in ALS patients with *SOD1* mutations [113]. Similarly, ASOs targeting *C9ORF72* repeat expansions mitigate RNA and protein toxicity, alleviating neuronal stress and restoring synaptic function [114]. Preclinical studies highlight that reducing mutant protein levels using gene silencing technologies can directly influence the neural E/I balance. In rodent models, ASO-mediated suppression of toxic *SOD1* reduced synaptic hyperexcitability and improved motor coordination, emphasizing the role of targeted genetic therapies in rebalancing neural circuits [115,116].

TDP-43 pathology, a hallmark of ALS and aging-related motor decline, involves nuclear depletion and cytoplasmic aggregation [117]. This leads to aberrant splicing of key target genes like *Stathmin-2* (*STMN2*), a regulator of axonal repair [117,118,119]. Specially, when TDP-43 binds to a GU-rich region in the *STMN2* pre-mRNA, it blocks the recognition of a cryptic 3’ splice site, resulting in aberrant splicing and reduced *STMN2* expression [118]. In turn, it impairs axonal regeneration and lysosomal trafficking, which are essential for maintaining neural excitability and homeostasis. CRISPR-Cas9 systems aimed at enhancing *STMN2* expression or correcting its splicing defects have been shown to restore axonal regeneration, demonstrating the interplay between gene function and neural E/I balance [118].

The translation of genetic therapies into clinical applications has gained momentum, with ongoing trials for ASOs targeting *SOD1* [120], *C9ORF72* [114], and *TDP-43* [121,122,123,124]. In addition to the above common ALS-related genes, the disruption of *REST* could cause ALS, which regulates neuroglobin (Ngb) expression in response to oxidative stress [125]. In ALS, the aggregation of mutant SOD1 and associated oxidative stress impair REST’s ability to protect neurons, further exacerbating cellular damage. Compromised regulation of neuroprotective pathways such as Ngb suggests that restoring REST function could help mitigate the effects of SOD1 mutations and reduce the neuronal damage that leads to E/I imbalance [125]. Furthermore, the autophagy-related pathway mediated by VPS-34 and the motor interneuron damage in the spinal cord caused by *STXBP1* deletion are both closely linked to motor aging. These discoveries have significantly reshaped our understanding of ALS, uncovering potential therapeutic avenues by targeting *REST*, *vps-34*, and *STXBP1*.

While the potential for using this approach is promising, gene editing technology is still experimental to restore E/I balance in aging brains [126]. Technical limitations, such as achieving precise, cell-specific delivery in the brain’s complex neural circuits and ensuring long-term stability of genetic modifications, are critical barriers, as even minor off-target effects could disrupt neural networks. Additionally, editing neuronal genomes raises debates about intergenerational consequences and the moral boundaries of human genetic modification. Concurrently, regulatory frameworks must evolve to address safety, efficacy, and ethical accountability in clinical use. While personalized approaches like patient-specific RNAi designs offer tailored solutions, their success hinges on overcoming technical bottlenecks, rigorous risk assessment, and establishing globally harmonized ethical guidelines [111,127].

### 5.2. Optogenetics

Optogenetics, a technique that uses light to control genetically modified neurons, has been employed to modulate neuronal activity precisely [128,129]. By activating or inhibiting specific neuronal populations, researchers can restore E/I balance, which may improve motor function and counteract age-related decline [128]. This enables targeted modulation of neuronal circuits, allowing researchers to counteract hyperexcitability by either dampening excitatory motor neurons or enhancing inhibitory GABAergic neurons to restore balance [130,131,132].

Osaki et al. demonstrate a novel approach using optogenetics integrated into a 3D ALS motor unit model [133]. This platform uses iPSC-derived motor neurons (MNs) and skeletal muscle fibers from ALS patients, cultured in a microfluidic device [133,134]. Through genetic modification, specific neurons are made to express light-sensitive ion channels, such as channelrhodopsins for excitation or halorhodopsins for inhibition [135]. When exposed to specific wavelengths of light, these channels either activate or inhibit neuronal firing. For example, blue light can excite neurons via channelrhodopsins, while yellow or green light can inhibit overactive neurons using halorhodopsins [135]. This experiment used channelrhodopsin-2 to precisely stimulate MNs to activate muscle contractions [134]. It provides an effective way to analyze NMJ function under ALS-like conditions. This innovative model provides a high-throughput platform for drug screening and elucidates ALS mechanisms by integrating optogenetics with patient-derived cells [133,136]. It demonstrates the critical interplay between motor neuron and muscle function, offering a pathway to identify therapeutic strategies for neurodegenerative diseases like ALS.

While optogenetics provides precise control over neuronal activity, it faces significant challenges when considering its translation to human therapies. These challenges include ethical and technical barriers, such as the need for invasive procedures to introduce light-sensitive proteins into target neurons and the implantation of optical fibers. Furthermore, the long-term effects of optogenetic manipulation on brain function remain largely unclear. Altering neuronal activity, particularly within motor circuits, comes with inherent risks, including potential seizures [137], motor impairments, or maladaptive plasticity [138]. Given these concerns, the risks associated with long-term optogenetic interventions in humans must be carefully evaluated to ensure the safety and efficacy of such therapies.

In brief, targeting the mechanisms that regulate synaptic excitation and inhibition holds promise for identifying novel therapeutic targets, such as synaptic proteins, receptor signaling pathways, or upstream genetic regulators critical for maintaining neural homeostasis. Current clinical strategies reflect this principle: Riluzole, the only globally approved disease-modifying therapy for ALS and a first-line agent per updated guidelines [139], exemplifies the multitargeted modulation of E/I balance. Its neuroprotective effects derive from sodium channel blockade, glutamate release reduction, and enhanced glutamate uptake [140]. However, the limited efficacy of purely glutamatergic agents (e.g., gabapentin, which fails to improve the survival of ALS) underscores the complexity of E/I dysregulation and the need for more precise interventions [141]. To address this, the integration of cutting-edge tools, such as optogenetics, chemogenetics, and precision gene editing (e.g., CRISPR-Cas9), offers tailored strategies to restore E/I balance [142,143]. These approaches not only enhance our understanding of synaptic and circuit dynamics but also pave the way for innovative treatments that could transform the management of ALS and other aging-related motor disorders (Figure 1B).

## 6. Conclusions

Maintaining a delicate balance between neural excitation and inhibition is critical for optimal nervous system function. Aging and genetic predispositions disrupt this equilibrium, contributing to the pathogenesis of motor neuron diseases. Emerging genetic-based therapies hold promise as innovative interventions, offering the potential to slow the progression of age-related motor diseases by targeting the electrical activity of excitatory and inhibitory neurons. Future research should focus on unraveling more precise molecular and cellular mechanisms underlying E/I imbalances, enabling the development of more targeted and effective therapeutic strategies. By addressing these foundational issues, the field can advance toward transformative treatments for ALS and related neurodegenerative conditions, despite the various challenges associated with human application.

## Figures and Tables

**Table 1:** Examples of the key genetic factors in E/I imbalance.

Gene	Feature	Function	Influence on E/I Balance	Reference
*SCN2A*	Ion channel	Encodes Nav1.2 sodium channel	Excitatory neural signal transmission	[32]
*CACNA1C*	Ion channel	Encodes voltage-dependent calcium channel (CaV1.2)	Allows calcium ions to flow into cells, and regulates the release of excitatory or inhibitory neurotransmitters	[33]
*GABRB3*	Ion channel	Encodes GABA_A_ receptor β3 subunit	Reduces neuronal excitability	[34]
*GRIN2A*	Glutamate receptor	Encodes NMDA receptor NR2A subunit	Excitatory signal transmission	[35]
*GRIN*	Glutamate receptor	Encodes subunits of NMDA receptors	Excitatory signal transmission	[36]
*SYT1*	Regulation of neurotransmitter release	Ca^2+^-triggered synaptic-binding protein release activator that mediates exocytosis	Promotes the release of excitatory or inhibitory neurotransmitters	[37]
*SYT2*	Regulation of neurotransmitter release	Regulates vesicle docking and fusion, synaptic vesicle retrieval	Promotes the release of excitatory or inhibitory neurotransmitters	[38]
*NRXNs*	Presynaptic adhesion protein	NRXN1: serotonin neurotransmitter regulator NLGN3: AMPA receptor cluster regulatory factor	Regulate the probability of presynaptic neurotransmitter release	[39]
*NLGNs*	Postsynaptic adhesion protein	Synaptic stabilizer; NRXN-NLGN interaction affects the function and efficiency of synaptic plasticity	Regulate the protein composition of the postsynaptic membrane and the probability of presynaptic neurotransmitter release	[40]
*TSC*	Signal transduction and regulatory factors	TSC-mTOR signaling pathway; regulates cell growth and synaptic plasticity	Enhances neuronal excitability	[41]
*PTEN*	Signal transduction and regulatory factors	Negative regulatory factor of PI3K/AKT pathway	Reduces the transmission of excitatory signals	[42]
*MECP2*	Signal transduction and regulatory factors	Methylated DNA-binding protein; regulates the development of the nervous system and neuronal function	Especially affects the function of GABAergic neurons	[43]
*BDNF*	Synaptic formation and plasticity-related proteins	Activates TrkB receptors	Regulates the development and function of GABAergic neurons	[44]
*UBE3A*	Protein degradation and ubiquitination system	Regulates nervous system protein degradation homeostasis	Maintains the balance of the E/I ratio	[45]

**Table 2:** A list of published genes related to E/I balance in motor aging.

Gene	Species	Mechanism	Influence for E/I Balance	Outcome	Reference
*REST*	Human	Decreases cortical activity by activating FOXO1 during aging	Decreases neuronal excitability	Longevity extension	[12]
	Mouse				
*spr-3* *spr-4*	*C. elegans*	Activate DAF-16-insulin/IGF-1 signaling pathway and repress ASH activity	Decrease neuronal excitability	Longevity extension	
*vps-34*	Mouse	Reduces neurotransmission at NMJ by inhibiting PI(3)P-PI-PI(4)P conversion	Reduces mPSC	Protect motor function in old age	[13]
	*C. elegans*				
*STXBP1*	Mouse	Protects PV and SST interneuron-mediated GABAergic synaptic transmission	Decreases neuronal excitability	Protects motor function	[14]

## Data Availability

Not applicable.
